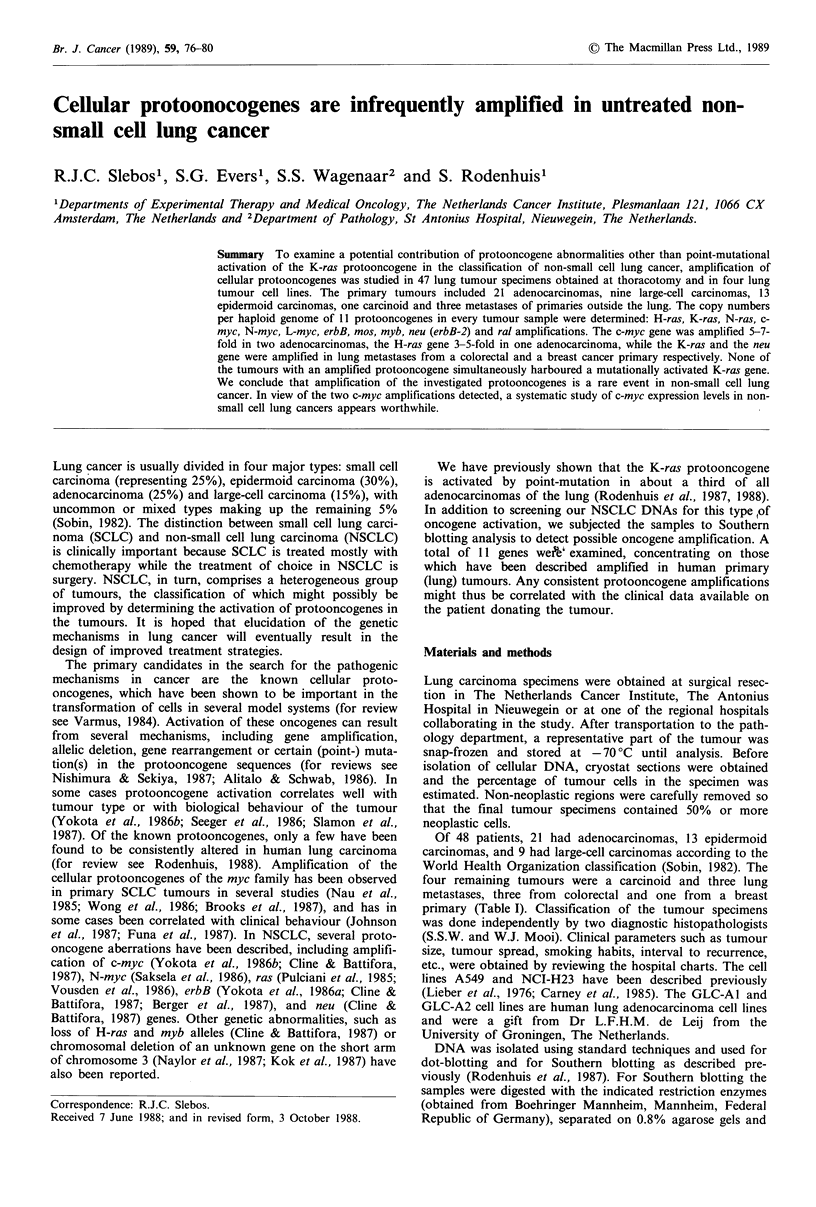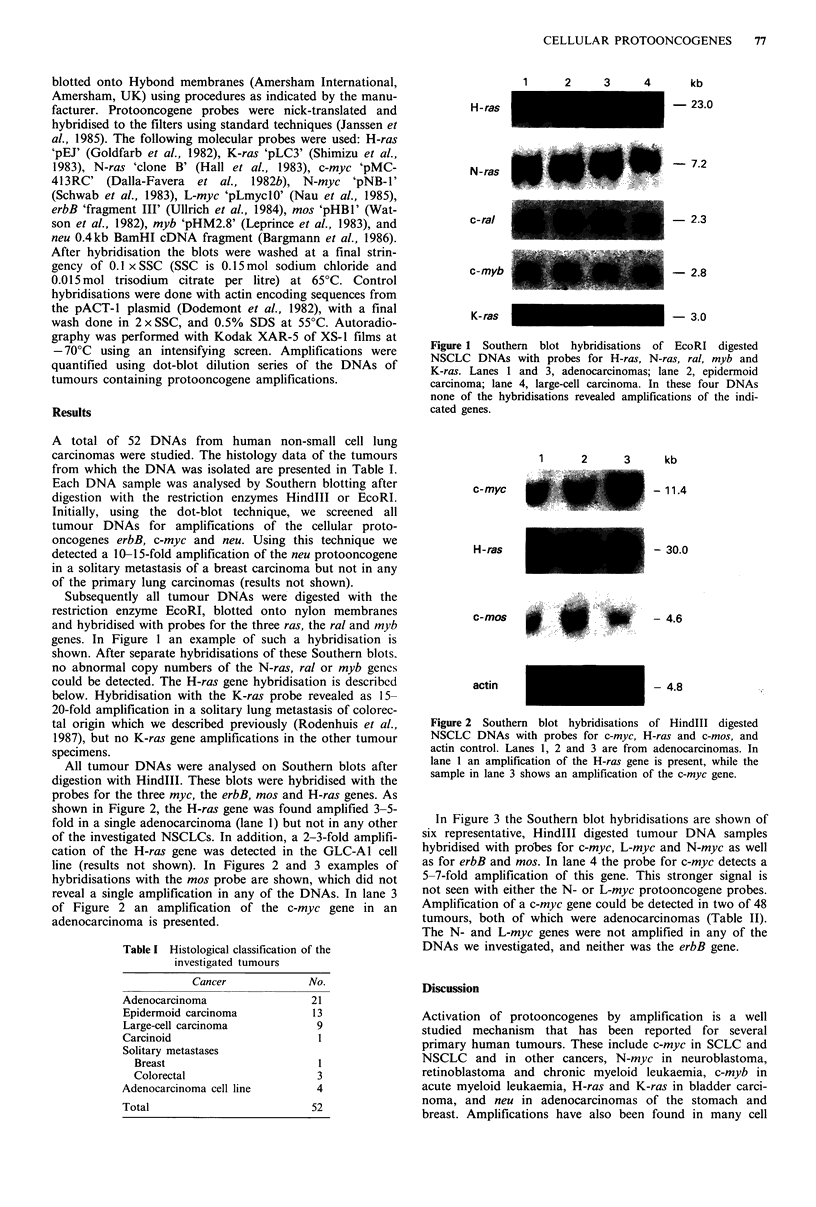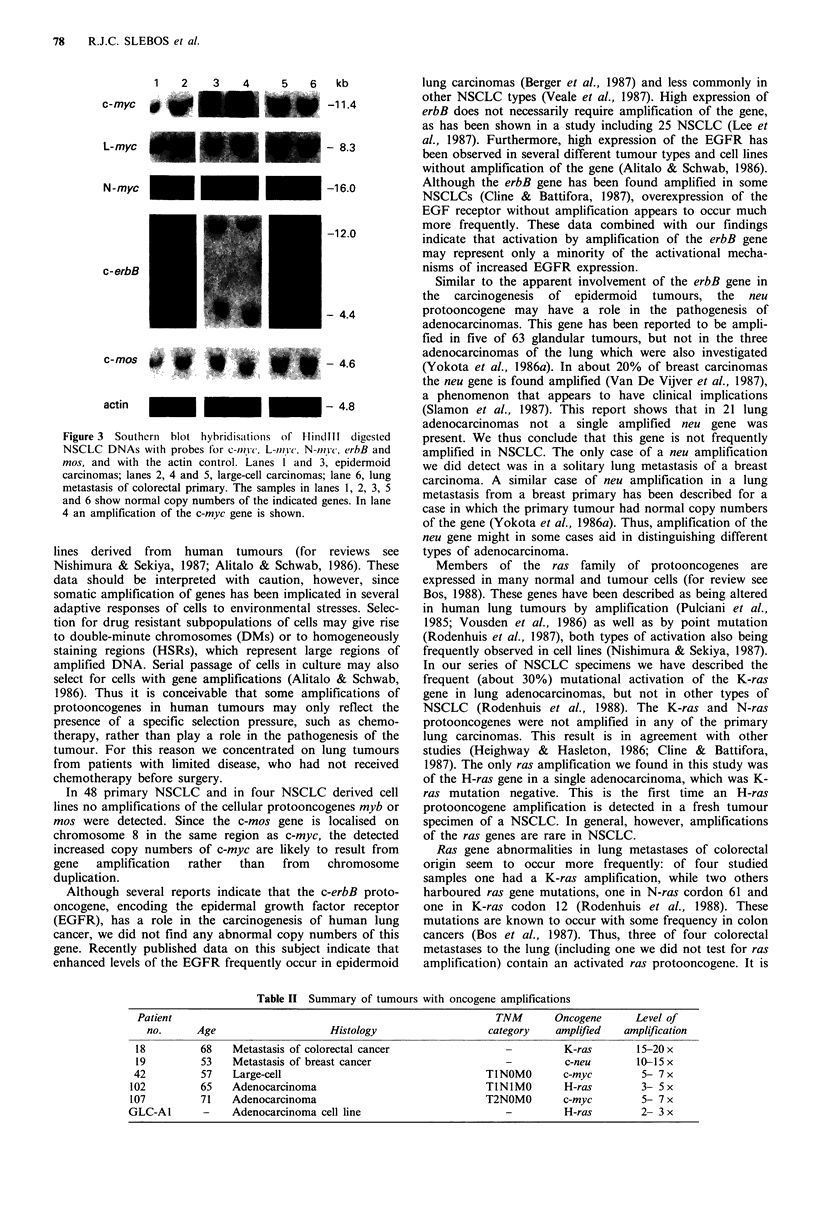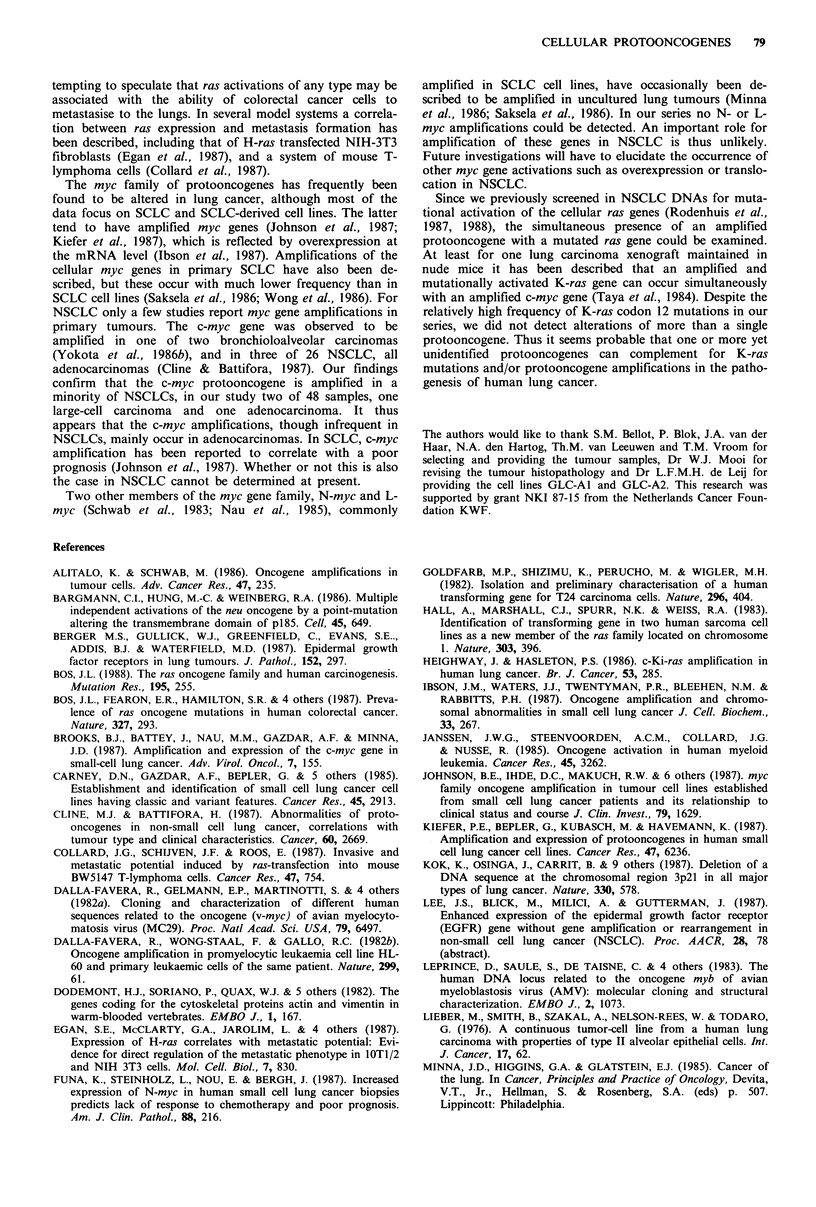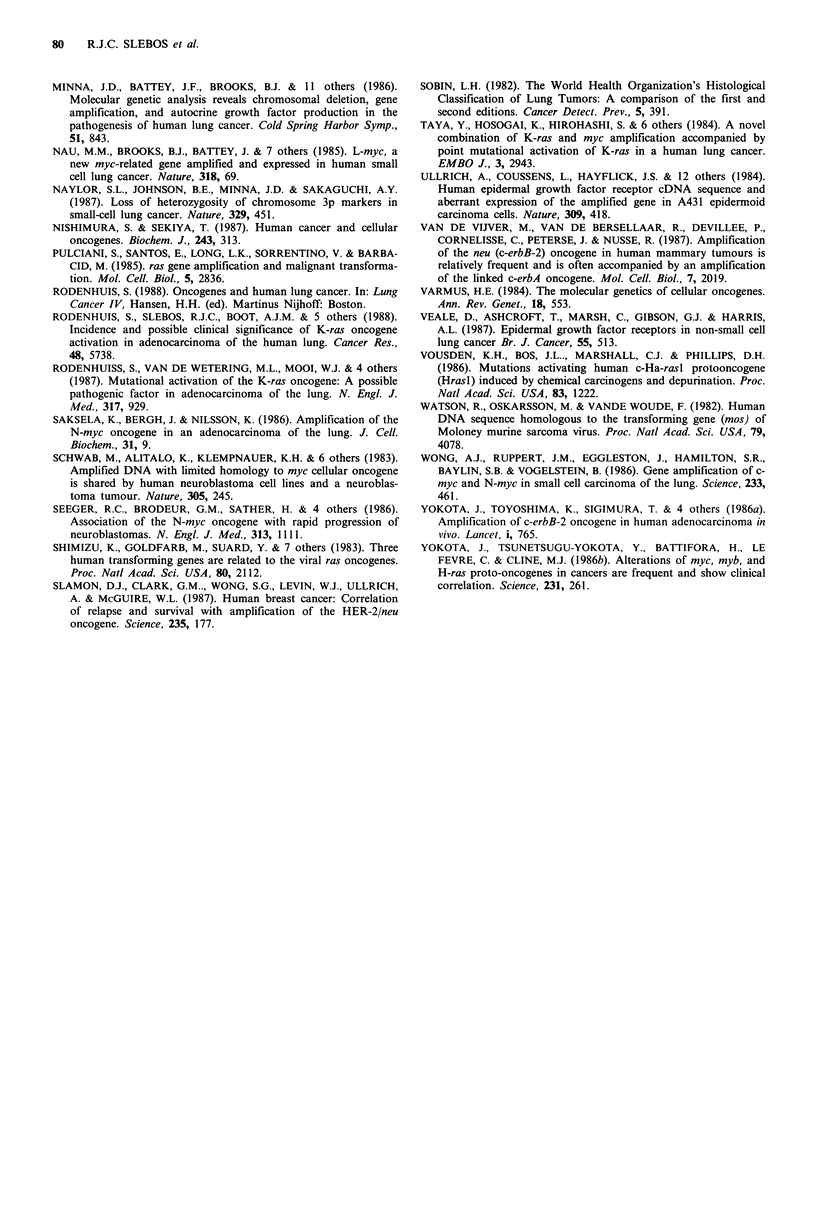# Cellular protoonocogenes are infrequently amplified in untreated non-small cell lung cancer.

**DOI:** 10.1038/bjc.1989.14

**Published:** 1989-01

**Authors:** R. J. Slebos, S. G. Evers, S. S. Wagenaar, S. Rodenhuis

**Affiliations:** Department of Experimental Therapy, Netherlands Cancer Institute, Amsterdam.

## Abstract

**Images:**


Br. J. Cancer (1989), 59, 76–80 © The Macmillan Press Ltd., 1989

### Cellular protoonocogenes are infrequently amplified in untreated non-small cell lung cancer

R.J.C. Slebos[1], S.G. Evers[1], S.S. Wagenaar[2] and S. Rodenhuis[1]

[1]*Departments of Experimental Therapy and Medical Oncology, The Netherlands Cancer Institute, Plesmanlaan 121, 1066 CX Amsterdam, The Netherlands and* [2]*Department of Pathology, St Antonius Hospital, Nieuwegein, The Netherlands.*

**Summary** To examine a potential contribution of protooncogene abnormalities other than point-mutational activation of the K-*ras* protooncogene in the classification of non-small cell lung cancer, amplification of cellular protooncogenes was studied in 47 lung tumour specimens obtained at thoracotomy and in four lung tumour cell lines. The primary tumours included 21 adenocarcinomas, nine large-cell carcinomas, 13 epidermoid carcinomas, one carcinoid and three metastases of primaries outside the lung. The copy numbers per haploid genome of 11 protooncogenes in every tumour sample were determined: H-*ras*, K-*ras*, N-*ras*, c-*myc*, N-*myc*, L-*myc*, *erbB*, *mos*, *myb*, *neu* (*erbB-2*) and *ral* amplifications. The c-*myc* gene was amplified 5–7-fold in two adenocarcinomas, the H-*ras* gene 3–5-fold in one adenocarcinoma, while the K-*ras* and the *neu* gene were amplified in lung metastases from a colorectal and a breast cancer primary respectively. None of the tumours with an amplified protooncogene simultaneously harboured a mutationally activated K-*ras* gene. We conclude that amplification of the investigated protooncogenes is a rare event in non-small cell lung cancer. In view of the two c-*myc* amplifications detected, a systematic study of c-*myc* expression levels in non-small cell lung cancers appears worthwhile.

Lung cancer is usually divided in four major types: small cell carcinoma (representing 25%), epidermoid carcinoma (30%), adenocarcinoma (25%) and large-cell carcinoma (15%), with uncommon or mixed types making up the remaining 5% (Sobin, 1982). The distinction between small cell lung carcinoma (SCLC) and non-small cell lung carcinoma (NSCLC) is clinically important because SCLC is treated mostly with chemotherapy while the treatment of choice in NSCLC is surgery. NSCLC, in turn, comprises a heterogeneous group of tumours, the classification of which might possibly be improved by determining the activation of protooncogenes in the tumours. It is hoped that elucidation of the genetic mechanisms in lung cancer will eventually result in the design of improved treatment strategies.

The primary candidates in the search for the pathogenic mechanisms in cancer are the known cellular protooncogenes, which have been shown to be important in the transformation of cells in several model systems (for review see Varmus, 1984). Activation of these oncogenes can result from several mechanisms, including gene amplification, allelic deletion, gene rearrangement or certain (point-) mutation(s) in the protooncogene sequences (for reviews see Nishimura & Sekiya, 1987; Alitalo & Schwab, 1986). In some cases protooncogene activation correlates well with tumour type or with biological behaviour of the tumour (Yokota *et al.*, 1986*b*; Seeger *et al.*, 1986; Slamon *et al.*, 1987). Of the known protooncogenes, only a few have been found to be consistently altered in human lung carcinoma (for review see Rodenhuis, 1988). Amplification of the cellular protooncogenes of the *myc* family has been observed in primary SCLC tumours in several studies (Nau *et al.*, 1985; Wong *et al.*, 1986; Brooks *et al.*, 1987), and has in some cases been correlated with clinical behaviour (Johnson *et al.*, 1987; Funa *et al.*, 1987). In NSCLC, several protooncogene aberrations have been described, including amplification of c-*myc* (Yokota *et al.*, 1986*b*; Cline & Battifora, 1987), N-*myc* (Saksela *et al.*, 1986), *ras* (Pulciani *et al.*, 1985; Vousden *et al.*, 1986), *erbB* (Yokota *et al.*, 1986*a*; Cline & Battifora, 1987; Berger *et al.*, 1987), and *neu* (Cline & Battifora, 1987) genes. Other genetic abnormalities, such as loss of H-*ras* and *myb* alleles (Cline & Battifora, 1987) or chromosomal deletion of an unknown gene on the short arm of chromosome 3 (Naylor *et al.*, 1987; Kok *et al.*, 1987) have also been reported.

Correspondence: R.J.C. Slebos.
Received 7 June 1988; and in revised form, 3 October 1988.

We have previously shown that the K-*ras* protooncogene is activated by point-mutation in about a third of all adenocarcinomas of the lung (Rodenhuis *et al.*, 1987, 1988). In addition to screening our NSCLC DNAs for this type of oncogene activation, we subjected the samples to Southern blotting analysis to detect possible oncogene amplification. A total of 11 genes were examined, concentrating on those which have been described amplified in human primary (lung) tumours. Any consistent protooncogene amplifications might thus be correlated with the clinical data available on the patient donating the tumour.

### Materials and methods

Lung carcinoma specimens were obtained at surgical resection in The Netherlands Cancer Institute, The Antonius Hospital in Nieuwegein or at one of the regional hospitals collaborating in the study. After transportation to the pathology department, a representative part of the tumour was snap-frozen and stored at $-70\,^{\circ}C$ until analysis. Before isolation of cellular DNA, cryostat sections were obtained and the percentage of tumour cells in the specimen was estimated. Non-neoplastic regions were carefully removed so that the final tumour specimens contained 50% or more neoplastic cells.

Of 48 patients, 21 had adenocarcinomas, 13 epidermoid carcinomas, and 9 had large-cell carcinomas according to the World Health Organization classification (Sobin, 1982). The four remaining tumours were a carcinoid and three lung metastases, three from colorectal and one from a breast primary (Table I). Classification of the tumour specimens was done independently by two diagnostic histopathologists (S.S.W. and W.J. Mooi). Clinical parameters such as tumour size, tumour spread, smoking habits, interval to recurrence, etc., were obtained by reviewing the hospital charts. The cell lines A549 and NCI-H23 have been described previously (Lieber *et al.*, 1976; Carney *et al.*, 1985). The GLC-A1 and GLC-A2 cell lines are human lung adenocarcinoma cell lines and were a gift from Dr L.F.H.M. de Leij from the University of Groningen, The Netherlands.

DNA was isolated using standard techniques and used for dot-blotting and for Southern blotting as described previously (Rodenhuis *et al.*, 1987). For Southern blotting the samples were digested with the indicated restriction enzymes (obtained from Boehringer Mannheim, Mannheim, Federal Republic of Germany), separated on 0.8% agarose gels and blotted onto Hybond membranes (Amersham International, Amersham, UK) using procedures as indicated by the manufacturer. Protooncogene probes were nick-translated and hybridised to the filters using standard techniques (Janssen *et al.*, 1985). The following molecular probes were used: H-*ras* 'pEJ' (Goldfarb *et al.*, 1982), K-*ras* 'pLC3' (Shimizu *et al.*, 1983), N-*ras* 'clone B' (Hall *et al.*, 1983), c-*myc* 'pMC-413RC' (Dalla-Favera *et al.*, 1982*b*), N-*myc* 'pNB-1' (Schwab *et al.*, 1983), L-*myc* 'pLmyc10' (Nau *et al.*, 1985), *erbB* 'fragment III' (Ullrich *et al.*, 1984), *mos* 'pHB1' (Watson *et al.*, 1982), *myb* 'pHM2.8' (Leprince *et al.*, 1983), and *neu* 0.4 kb BamHI cDNA fragment (Bargmann *et al.*, 1986). After hybridisation the blots were washed at a final stringency of 0.1 × SSC (SSC is 0.15 mol sodium chloride and 0.015 mol trisodium citrate per litre) at 65°C. Control hybridisations were done with actin encoding sequences from the pACT-1 plasmid (Dodemont *et al.*, 1982), with a final wash done in 2 × SSC, and 0.5% SDS at 55°C. Autoradiography was performed with Kodak XAR-5 of XS-1 films at −70°C using an intensifying screen. Amplifications were quantified using dot-blot dilution series of the DNAs of tumours containing protooncogene amplifications.

### Results

A total of 52 DNAs from human non-small cell lung carcinomas were studied. The histology data of the tumours from which the DNA was isolated are presented in Table I. Each DNA sample was analysed by Southern blotting after digestion with the restriction enzymes HindIII or EcoRI. Initially, using the dot-blot technique, we screened all tumour DNAs for amplifications of the cellular protooncogenes *erbB*, c-*myc* and *neu*. Using this technique we detected a 10–15-fold amplification of the *neu* protooncogene in a solitary metastasis of a breast carcinoma but not in any of the primary lung carcinomas (results not shown).

Subsequently all tumour DNAs were digested with the restriction enzyme EcoRI, blotted onto nylon membranes and hybridised with probes for the three *ras*, the *ral* and *myb* genes. In Figure 1 an example of such a hybridisation is shown. After separate hybridisations of these Southern blots, no abnormal copy numbers of the N-*ras*, *ral* or *myb* genes could be detected. The H-*ras* gene hybridisation is described below. Hybridisation with the K-*ras* probe revealed as 15–20-fold amplification in a solitary lung metastasis of colorectal origin which we described previously (Rodenhuis *et al.*, 1987), but no K-*ras* gene amplifications in the other tumour specimens.

All tumour DNAs were analysed on Southern blots after digestion with HindIII. These blots were hybridised with the probes for the three *myc*, the *erbB*, *mos* and H-*ras* genes. As shown in Figure 2, the H-*ras* gene was found amplified 3–5-fold in a single adenocarcinoma (lane 1) but not in any other of the investigated NSCLCs. In addition, a 2–3-fold amplification of the H-*ras* gene was detected in the GLC-A1 cell line (results not shown). In Figures 2 and 3 examples of hybridisations with the *mos* probe are shown, which did not reveal a single amplification in any of the DNAs. In lane 3 of Figure 2 an amplification of the c-*myc* gene in an adenocarcinoma is presented.

**Table I** Histological classification of the investigated tumours

| *Cancer* | *No.* |
|---|---|
| Adenocarcinoma | 21 |
| Epidermoid carcinoma | 13 |
| Large-cell carcinoma | 9 |
| Carcinoid | 1 |
| Solitary metastases | |
| Breast | 1 |
| Colorectal | 3 |
| Adenocarcinoma cell line | 4 |
| Total | 52 |

**Figure 1** Southern blot hybridisations of EcoRI digested NSCLC DNAs with probes for H-*ras*, N-*ras*, *ral*, *myb* and K-*ras*. Lanes 1 and 3, adenocarcinomas; lane 2, epidermoid carcinoma; lane 4, large-cell carcinoma. In these four DNAs none of the hybridisations revealed amplifications of the indicated genes.

**Figure 2** Southern blot hybridisations of HindIII digested NSCLC DNAs with probes for c-*myc*, H-*ras* and c-*mos*, and actin control. Lanes 1, 2 and 3 are from adenocarcinomas. In lane 1 an amplification of the H-*ras* gene is present, while the sample in lane 3 shows an amplification of the c-*myc* gene.

In Figure 3 the Southern blot hybridisations are shown of six representative, HindIII digested tumour DNA samples hybridised with probes for c-*myc*, L-*myc* and N-*myc* as well as for *erbB* and *mos*. In lane 4 the probe for c-*myc* detects a 5–7-fold amplification of this gene. This stronger signal is not seen with either the N- or L-*myc* protooncogene probes. Amplification of a c-*myc* gene could be detected in two of 48 tumours, both of which were adenocarcinomas (Table II). The N- and L-*myc* genes were not amplified in any of the DNAs we investigated, and neither was the *erbB* gene.

### Discussion

Activation of protooncogenes by amplification is a well studied mechanism that has been reported for several primary human tumours. These include c-*myc* in SCLC and NSCLC and in other cancers, N-*myc* in neuroblastoma, retinoblastoma and chronic myeloid leukaemia, c-*myb* in acute myeloid leukaemia, H-*ras* and K-*ras* in bladder carcinoma, and *neu* in adenocarcinomas of the stomach and breast. Amplifications have also been found in many cell

**Figure 3** Southern blot hybridisations of HindIII digested NSCLC DNAs with probes for c-*myc*, L-*myc*, N-*myc*, *erbB* and *mos*, and with the actin control. Lanes 1 and 3, epidermoid carcinomas; lanes 2, 4 and 5, large-cell carcinomas; lane 6, lung metastasis of colorectal primary. The samples in lanes 1, 2, 3, 5 and 6 show normal copy numbers of the indicated genes. In lane 4 an amplification of the c-*myc* gene is shown.

lines derived from human tumours (for reviews see Nishimura & Sekiya, 1987; Alitalo & Schwab, 1986). These data should be interpreted with caution, however, since somatic amplification of genes has been implicated in several adaptive responses of cells to environmental stresses. Selection for drug resistant subpopulations of cells may give rise to double-minute chromosomes (DMs) or to homogeneously staining regions (HSRs), which represent large regions of amplified DNA. Serial passage of cells in culture may also select for cells with gene amplifications (Alitalo & Schwab, 1986). Thus it is conceivable that some amplifications of protooncogenes in human tumours may only reflect the presence of a specific selection pressure, such as chemotherapy, rather than play a role in the pathogenesis of the tumour. For this reason we concentrated on lung tumours from patients with limited disease, who had not received chemotherapy before surgery.

In 48 primary NSCLC and in four NSCLC derived cell lines no amplifications of the cellular protooncogenes *myb* or *mos* were detected. Since the c-*mos* gene is localised on chromosome 8 in the same region as c-*myc*, the detected increased copy numbers of c-*myc* are likely to result from gene amplification rather than from chromosome duplication.

Although several reports indicate that the c-*erbB* protooncogene, encoding the epidermal growth factor receptor (EGFR), has a role in the carcinogenesis of human lung cancer, we did not find any abnormal copy numbers of this gene. Recently published data on this subject indicate that enhanced levels of the EGFR frequently occur in epidermoid lung carcinomas (Berger *et al.*, 1987) and less commonly in other NSCLC types (Veale *et al.*, 1987). High expression of *erbB* does not necessarily require amplification of the gene, as has been shown in a study including 25 NSCLC (Lee *et al.*, 1987). Furthermore, high expression of the EGFR has been observed in several different tumour types and cell lines without amplification of the gene (Alitalo & Schwab, 1986). Although the *erbB* gene has been found amplified in some NSCLCs (Cline & Battifora, 1987), overexpression of the EGF receptor without amplification appears to occur much more frequently. These data combined with our findings indicate that activation by amplification of the *erbB* gene may represent only a minority of the activational mechanisms of increased EGFR expression.

Similar to the apparent involvement of the *erbB* gene in the carcinogenesis of epidermoid tumours, the *neu* protooncogene may have a role in the pathogenesis of adenocarcinomas. This gene has been reported to be amplified in five of 63 glandular tumours, but not in the three adenocarcinomas of the lung which were also investigated (Yokota *et al.*, 1986*a*). In about 20% of breast carcinomas the *neu* gene is found amplified (Van De Vijver *et al.*, 1987), a phenomenon that appears to have clinical implications (Slamon *et al.*, 1987). This report shows that in 21 lung adenocarcinomas not a single amplified *neu* gene was present. We thus conclude that this gene is not frequently amplified in NSCLC. The only case of a *neu* amplification we did detect was in a solitary lung metastasis of a breast carcinoma. A similar case of *neu* amplification in a lung metastasis from a breast primary has been described for a case in which the primary tumour had normal copy numbers of the gene (Yokota *et al.*, 1986*a*). Thus, amplification of the *neu* gene might in some cases aid in distinguishing different types of adenocarcinoma.

Members of the *ras* family of protooncogenes are expressed in many normal and tumour cells (for review see Bos, 1988). These genes have been described as being altered in human lung tumours by amplification (Pulciani *et al.*, 1985; Vousden *et al.*, 1986) as well as by point mutation (Rodenhuis *et al.*, 1987), both types of activation also being frequently observed in cell lines (Nishimura & Sekiya, 1987). In our series of NSCLC specimens we have described the frequent (about 30%) mutational activation of the K-*ras* gene in lung adenocarcinomas, but not in other types of NSCLC (Rodenhuis *et al.*, 1988). The K-*ras* and N-*ras* protooncogenes were not amplified in any of the primary lung carcinomas. This result is in agreement with other studies (Heighway & Hasleton, 1986; Cline & Battifora, 1987). The only *ras* amplification we found in this study was of the H-*ras* gene in a single adenocarcinoma, which was K-*ras* mutation negative. This is the first time an H-*ras* protooncogene amplification is detected in a fresh tumour specimen of a NSCLC. In general, however, amplifications of the *ras* genes are rare in NSCLC.

*Ras* gene abnormalities in lung metastases of colorectal origin seem to occur more frequently: of four studied samples one had a K-*ras* amplification, while two others harboured *ras* gene mutations, one in N-*ras* cordon 61 and one in K-*ras* codon 12 (Rodenhuis *et al.*, 1988). These mutations are known to occur with some frequency in colon cancers (Bos *et al.*, 1987). Thus, three of four colorectal metastases to the lung (including one we did not test for *ras* amplification) contain an activated *ras* protooncogene. It is

**Table II** Summary of tumours with oncogene amplifications

| *Patient no.* | *Age* | *Histology* | *TNM category* | *Oncogene amplified* | *Level of amplification* |
|---|---|---|---|---|---|
| 18 | 68 | Metastasis of colorectal cancer | – | K-*ras* | 15–20 × |
| 19 | 53 | Metastasis of breast cancer | – | c-*neu* | 10–15 × |
| 42 | 57 | Large-cell | T1N0M0 | c-*myc* | 5– 7 × |
| 102 | 65 | Adenocarcinoma | T1N1M0 | H-*ras* | 3– 5 × |
| 107 | 71 | Adenocarcinoma | T2N0M0 | c-*myc* | 5– 7 × |
| GLC-A1 | – | Adenocarcinoma cell line | – | H-*ras* | 2– 3 × |

tempting to speculate that *ras* activations of any type may be associated with the ability of colorectal cancer cells to metastasise to the lungs. In several model systems a correlation between *ras* expression and metastasis formation has been described, including that of H-*ras* transfected NIH-3T3 fibroblasts (Egan *et al.*, 1987), and a system of mouse T-lymphoma cells (Collard *et al.*, 1987).

The *myc* family of protooncogenes has frequently been found to be altered in lung cancer, although most of the data focus on SCLC and SCLC-derived cell lines. The latter tend to have amplified *myc* genes (Johnson *et al.*, 1987; Kiefer *et al.*, 1987), which is reflected by overexpression at the mRNA level (Ibson *et al.*, 1987). Amplifications of the cellular *myc* genes in primary SCLC have also been described, but these occur with much lower frequency than in SCLC cell lines (Saksela *et al.*, 1986; Wong *et al.*, 1986). For NSCLC only a few studies report *myc* gene amplifications in primary tumours. The c-*myc* gene was observed to be amplified in one of two bronchioloalveolar carcinomas (Yokota *et al.*, 1986*b*), and in three of 26 NSCLC, all adenocarcinomas (Cline & Battifora, 1987). Our findings confirm that the c-*myc* protooncogene is amplified in a minority of NSCLCs, in our study two of 48 samples, one large-cell carcinoma and one adenocarcinoma. It thus appears that the c-*myc* amplifications, though infrequent in NSCLCs, mainly occur in adenocarcinomas. In SCLC, c-*myc* amplification has been reported to correlate with a poor prognosis (Johnson *et al.*, 1987). Whether or not this is also the case in NSCLC cannot be determined at present.

Two other members of the *myc* gene family, N-*myc* and L-*myc* (Schwab *et al.*, 1983; Nau *et al.*, 1985), commonly amplified in SCLC cell lines, have occasionally been described to be amplified in uncultured lung tumours (Minna *et al.*, 1986; Saksela *et al.*, 1986). In our series no N- or L-*myc* amplifications could be detected. An important role for amplification of these genes in NSCLC is thus unlikely. Future investigations will have to elucidate the occurrence of other *myc* gene activations such as overexpression or translocation in NSCLC.

Since we previously screened in NSCLC DNAs for mutational activation of the cellular *ras* genes (Rodenhuis *et al.*, 1987, 1988), the simultaneous presence of an amplified protooncogene with a mutated *ras* gene could be examined. At least for one lung carcinoma xenograft maintained in nude mice it has been described that an amplified and mutationally activated K-*ras* gene can occur simultaneously with an amplified c-*myc* gene (Taya *et al.*, 1984). Despite the relatively high frequency of K-*ras* codon 12 mutations in our series, we did not detect alterations of more than a single protooncogene. Thus it seems probable that one or more yet unidentified protooncogenes can complement for K-*ras* mutations and/or protooncogene amplifications in the pathogenesis of human lung cancer.

The authors would like to thank S.M. Bellot, P. Blok, J.A. van der Haar, N.A. den Hartog, Th.M. van Leeuwen and T.M. Vroom for selecting and providing the tumour samples, Dr W.J. Mooi for revising the tumour histopathology and Dr L.F.M.H. de Leij for providing the cell lines GLC-A1 and GLC-A2. This research was supported by grant NKI 87-15 from the Netherlands Cancer Foundation KWF.

### References

ALITALO, K. & SCHWAB, M. (1986). Oncogene amplifications in tumour cells. *Adv. Cancer Res.*, **47**, 235.

BARGMANN, C.I., HUNG, M.-C. & WEINBERG, R.A. (1986). Multiple independent activations of the *neu* oncogene by a point-mutation altering the transmembrane domain of p185. *Cell*, **45**, 649.

BERGER M.S., GULLICK, W.J., GREENFIELD, C., EVANS, S.E.., ADDIS, B.J. & WATERFIELD, M.D. (1987). Epidermal growth factor receptors in lung tumours. *J. Pathol.*, **152**, 297.

BOS, J.L. (1988). The *ras* oncogene family and human carcinogenesis. *Mutation Res.*, **195**, 255.

BOS, J.L., FEARON, E.R., HAMILTON, S.R. & 4 others (1987). Prevalence of *ras* oncogene mutations in human colorectal cancer. *Nature*, **327**, 293.

BROOKS, B.J., BATTEY, J., NAU, M.M., GAZDAR, A.F. & MINNA, J.D. (1987). Amplification and expression of the c-*myc* gene in small-cell lung cancer. *Adv. Virol. Oncol.*, **7**, 155.

CARNEY, D.N., GAZDAR, A.F., BEPLER, G. & 5 others (1985). Establishment and identification of small cell lung cancer cell lines having classic and variant features. *Cancer Res.*, **45**, 2913.

CLINE, M.J. & BATTIFORA, H. (1987). Abnormalities of protooncogenes in non-small cell lung cancer, correlations with tumour type and clinical characteristics. *Cancer*, **60**, 2669.

COLLARD, J.G., SCHIJVEN, J.F. & ROOS, E. (1987). Invasive and metastatic potential induced by *ras*-transfection into mouse BW5147 T-lymphoma cells. *Cancer Res.*, **47**, 754.

DALLA-FAVERA, R., GELMANN, E.P., MARTINOTTI, S. & 4 others (1982*a*). Cloning and characterization of different human sequences related to the oncogene (v-*myc)* of avian myelocytomatosis virus (MC29). *Proc. Natl Acad. Sci. USA*, **79**, 6497.

DALLA-FAVERA, R., WONG-STAAL, F. & GALLO, R.C. (1982*b*). Oncogene amplification in promyelocytic leukaemia cell line HL-60 and primary leukaemic cells of the same patient. *Nature*, **299**, 61.

DODEMONT, H.J., SORIANO, P., QUAX, W.J. & 5 others (1982). The genes coding for the cytoskeletal proteins actin and vimentin in warm-blooded vertebrates. *EMBO J.*, **1**, 167.

EGAN, S.E., McCLARTY, G.A., JAROLIM, L. & 4 others (1987). Expression of H-*ras* correlates with metastatic potential: Evidence for direct regulation of the metastatic phenotype in 10T1/2 and NIH 3T3 cells. *Mol. Cell. Biol.*, **7**, 830.

FUNA, K., STEINHOLZ, L., NOU, E. & BERGH, J. (1987). Increased expression of N-*myc* in human small cell lung cancer biopsies predicts lack of response to chemotherapy and poor prognosis. *Am. J. Clin. Pathol.*, **88**, 216.

GOLDFARB, M.P., SHIZIMU, K., PERUCHO, M. & WIGLER, M.H. (1982). Isolation and preliminary characterisation of a human transforming gene for T24 carcinoma cells. *Nature*, **296**, 404.

HALL, A., MARSHALL, C.J., SPURR, N.K. & WEISS, R.A. (1983). Identification of transforming gene in two human sarcoma cell lines as a new member of the *ras* family located on chromosome 1. *Nature*, **303**, 396.

HEIGHWAY, J. & HASLETON, P.S. (1986). c-Ki-*ras* amplification in human lung cancer. *Br. J. Cancer*, **53**, 285.

IBSON, J.M., WATERS, J.J., TWENTYMAN, P.R., BLEEHEN, N.M. & RABBITTS, P.H. (1987). Oncogene amplification and chromosomal abnormalities in small cell lung cancer *J. Cell. Biochem.*, **33**, 267.

JANSSEN, J.W.G., STEENVOORDEN, A.C.M., COLLARD, J.G. & NUSSE, R. (1985). Oncogene activation in human myeloid leukemia. *Cancer Res.*, **45**, 3262.

JOHNSON, B.E., IHDE, D.C., MAKUCH, R.W. & 6 others (1987). *myc* family oncogene amplification in tumour cell lines established from small cell lung cancer patients and its relationship to clinical status and course *J. Clin. Invest.*, **79**, 1629.

KIEFER, P.E., BEPLER, G., KUBASCH, M. & HAVEMANN, K. (1987). Amplification and expression of protooncogenes in human small cell lung cancer cell lines. *Cancer Res.*, **47**, 6236.

KOK, K., OSINGA, J., CARRIT, B. & 9 others (1987). Deletion of a DNA sequence at the chromosomal region 3p21 in all major types of lung cancer. *Nature*, **330**, 578.

LEE, J.S., BLICK, M., MILICI, A. & GUTTERMAN, J. (1987). Enhanced expression of the epidermal growth factor receptor (EGFR) gene without gene amplification or rearrangement in non-small cell lung cancer (NSCLC). *Proc. AACR*, **28**, 78 (abstract).

LEPRINCE, D., SAULE, S., DE TAISNE, C. & 4 others (1983). The human DNA locus related to the oncogene *myb* of avian myeloblastosis virus (AMV): molecular cloning and structural characterization. *EMBO J.*, **2**, 1073.

LIEBER, M., SMITH, B., SZAKAL, A., NELSON-REES, W. & TODARO, G. (1976). A continuous tumor-cell line from a human lung carcinoma with properties of type II alveolar epithelial cells. *Int. J. Cancer*, **17**, 62.

MINNA, J.D., HIGGINS, G.A. & GLATSTEIN, E.J. (1985). Cancer of the lung. In *Cancer, Principles and Practice of Oncology*, Devita, V.T., Jr., Hellman, S. & Rosenberg, S.A. (eds) p. 507. Lippincott: Philadelphia.

MINNA, J.D., BATTEY, J.F., BROOKS, B.J. & 11 others (1986). Molecular genetic analysis reveals chromosomal deletion, gene amplification, and autocrine growth factor production in the pathogenesis of human lung cancer. *Cold Spring Harbor Symp.*, **51**, 843.

NAU, M.M., BROOKS, B.J., BATTEY, J. & 7 others (1985). L-*myc*, a new *myc*-related gene amplified and expressed in human small cell lung cancer. *Nature*, **318**, 69.

NAYLOR, S.L., JOHNSON, B.E., MINNA, J.D. & SAKAGUCHI, A.Y. (1987). Loss of heterozygosity of chromosome 3p markers in small-cell lung cancer. *Nature*, **329**, 451.

NISHIMURA, S. & SEKIYA, T. (1987). Human cancer and cellular oncogenes. *Biochem. J.*, **243**, 313.

PULCIANI, S., SANTOS, E., LONG, L.K., SORRENTINO, V. & BARBACID, M. (1985). *ras* gene amplification and malignant transformation. *Mol. Cell. Biol.*, **5**, 2836.

RODENHUIS, S. (1988). Oncogenes and human lung cancer. In: *Lung Cancer IV*, Hansen, H.H. (ed). Martinus Nijhoff: Boston.

RODENHUIS, S., SLEBOS, R.J.C., BOOT, A.J.M. & 5 others (1988). Incidence and possible clinical significance of K-*ras* oncogene activation in adenocarcinoma of the human lung. *Cancer Res.*, **48**, 5738.

RODENHUISS, S., VAN DE WETERING, M.L., MOOI, W.J. & 4 others (1987). Mutational activation of the K-*ras* oncogene: A possible pathogenic factor in adenocarcinoma of the lung. *N. Engl. J. Med.*, **317**, 929.

SAKSELA, K., BERGH, J. & NILSSON, K. (1986). Amplification of the N-*myc* oncogene in an adenocarcinoma of the lung. *J. Cell. Biochem.*, **31**, 9.

SCHWAB, M., ALITALO, K., KLEMPNAUER, K.H. & 6 others (1983). Amplified DNA with limited homology to *myc* cellular oncogene is shared by human neuroblastoma cell lines and a neuroblastoma tumour. *Nature*, **305**, 245.

SEEGER, R.C., BRODEUR, G.M., SATHER, H. & 4 others (1986). Association of the N-*myc* oncogene with rapid progression of neuroblastomas. *N. Engl. J. Med.*, **313**, 1111.

SHIMIZU, K., GOLDFARB, M., SUARD, Y. & 7 others (1983). Three human transforming genes are related to the viral *ras* oncogenes. *Proc. Natl Acad. Sci. USA*, **80**, 2112.

SLAMON, D.J., CLARK, G.M.., WONG, S.G., LEVIN, W.J., ULLRICH, A. & McGUIRE, W.L. (1987). Human breast cancer: Correlation of relapse and survival with amplification of the HER-2/*neu* oncogene. *Science*, **235**, 177.

SOBIN, L.H. (1982). The World Health Organization's Histological Classification of Lung Tumors: A comparison of the first and second editions. *Cancer Detect. Prev.*, **5**, 391.

TAYA, Y., HOSOGAI, K., HIROHASHI, S. & 6 others (1984). A novel combination of K-*ras* and *myc* amplification accompanied by point mutational activation of K-*ras* in a human lung cancer. *EMBO J.*, **3**, 2943.

ULLRICH, A., COUSSENS, L., HAYFLICK, J.S. & 12 others (1984). Human epidermal growth factor receptor cDNA sequence and aberrant expression of the amplified gene in A431 epidermoid carcinoma cells. *Nature*, **309**, 418.

VAN DE VIJVER, M., VAN DE BERSELLAAR, R., DEVILLEE, P., CORNELISSE, C., PETERSE, J. & NUSSE, R. (1987). Amplification of the *neu* (c-*erbB*-2) oncogene in human mammary tumours is relatively frequent and is often accompanied by an amplification of the linked c-*erbA* oncogene. *Mol. Cell. Biol.*, **7**, 2019.

VARMUS, H.E. (1984). The molecular genetics of cellular oncogenes. *Ann. Rev. Genet.*, **18**, 553.

VEALE, D., ASHCROFT, T., MARSH, C., GIBSON, G.J. & HARRIS, A.L. (1987). Epidermal growth factor receptors in non-small cell lung cancer *Br. J. Cancer*, **55**, 513.

VOUSDEN, K.H., BOS, J.L.., MARSHALL, C.J. & PHILLIPS, D.H. (1986). Mutations activating human c-Ha-*ras*1 protooncogene (H*ras*1) induced by chemical carcinogens and depurination. *Proc. Natl Acad. Sci. USA*, **83**, 1222.

WATSON, R., OSKARSSON, M. & VANDE WOUDE, F. (1982). Human DNA sequence homologous to the transforming gene (*mos*) of Moloney murine sarcoma virus. *Proc. Natl Acad. Sci. USA*, **79**, 4078.

WONG, A.J., RUPPERT, J.M., EGGLESTON, J., HAMILTON, S.R., BAYLIN, S.B. & VOGELSTEIN, B. (1986). Gene amplification of c-*myc* and N-*myc* in small cell carcinoma of the lung. *Science*, **233**, 461.

YOKOTA, J., TOYOSHIMA, K., SIGIMURA, T. & 4 others (1986*a*). Amplification of c-*erbB*-2 oncogene in human adenocarcinoma *in vivo*. *Lancet*, **i**, 765.

YOKOTA, J., TSUNETSUGU-YOKOTA, Y., BATTIFORA, H., LE FEVRE, C. & CLINE, M.J. (1986*b*). Alterations of *myc*, *myb*, and H-*ras* proto-oncogenes in cancers are frequent and show clinical correlation. *Science*, **231**, 261.